# Adverse outcomes of artificial pneumothorax under right bronchial occlusion for patients with thoracoscopic-assisted oesophagectomy in the prone position versus the semiprone position

**DOI:** 10.3389/fonc.2022.919910

**Published:** 2022-08-09

**Authors:** Qiongzhen Li, Mingye Zhao, Dongjin Wu, Xufeng Guo, Jingxiang Wu

**Affiliations:** ^1^ Department of Anesthesiology, Shanghai Chest Hospital, Shanghai Jiaotong University, Shanghai, China; ^2^ Department of Thoracic Surgery, Shanghai Chest Hospital, Shanghai Jiaotong University, Shanghai, China

**Keywords:** oesophagectomy, thoracoscopy, artificial pneumothorax, one-lung ventilation (OLV), prone position, semiprone position

## Abstract

**Background:**

There are few studies on the impact of body position on variations in circulation and breathing, and it has not been confirmed whether body position changes can reduce the pulmonary complications of thoracoscopic-assisted oesophagectomy.

**Methods:**

A single-center retrospective study included patients undergoing thoracoscopic-assisted oesophagectomy in the prone position or semiprone position between 1 July 2020, and 30 June 2021, at the Shanghai Chest Hospital. There were 103 patients with thoracoscopic-assisted oesophagectomy in the final analysis, including 43 patients undergoing thoracoscopic-assisted oesophagectomy in the prone position. Postoperative pulmonary complication (PPC) incidence was the primary endpoint. The incidence of cardiovascular and other complications was the secondary endpoint. Chest tube duration, patient-controlled anaesthesia (PCA) pressing frequency within 24 h, ICU stay, and the postoperative hospital length of stay (LOS) were also collected.

**Results:**

Compared with the semiprone position, the prone position decreased the incidence of atelectasis (12% vs. 30%, P = 0.032). Nevertheless, there were no considerable differences in the rates of cardiovascular and other complications, ICU stay, or LOS (P >0.05). Multivariable logistic regression analysis showed that the prone position (OR = 0.196, P = 0.011), no smoking (OR = 0.103, P <0.001), preoperative DLCO% ≥90% (OR = 0.230, P = 0.003), and an operative time <180 min (OR = 0.268, P = 0.006) were associated with less atelectasis.

**Conclusions:**

Our study shows that artificial pneumothorax under right bronchial occlusion one-lung ventilation for patients with thoracoscopic-assisted oesophagectomy in the prone position can decrease postoperative atelectasis compared with the semiprone position.

## 1 Introduction

Oesophagectomy with thoracoscopic assistance is a lengthy and traumatising procedure. Postoperative pulmonary complications (PPCs) are the most common serious complications after this type of surgery. PPCs are major causes of morbidity or mortality, prolonged hospital stays and added resource use (1–3). An extensive patient, anaesthetic, and surgical elements are related to PPCs (4).

Some studies have indicated that the prone position decreases postoperative respiratory complications (5, 6) with technical merit. This approach has many advantages, including good oxygen saturation (7).The prone position for patients having oesophageal cancer was found to be secure and effective. Additionally, the prone position might be a less invasive manoeuvre than the semiprone position (8–12).

It is suggested that artificial pneumothorax under two-lung ventilation is beneficial for maintaining steady haemodynamics and oxygenation in thoracoscopic-assisted oesophagectomy in the prone position (13). Despite common anaesthetic administration with one-lung ventilation, prone position-based thoracoscopic-assisted oesophagectomy has been popular as it can guarantee the operative field and manoeuvrability. The demands for mastery extend beyond surgical steps to anaesthetic administration and surgical nursing during the introduction of prone position surgery.

At our hospital, under right bronchial occlusion, one-lung ventilation-based prone position oesophagectomy was adopted to create artificial pneumothorax in 43 cases from July 2020 to June 2021. Since 2020, surgery has been performed with artificial pneumothorax pressure and gravity-based exclusion of the lungs from the operative field under a single lumen tube-based anaesthetic administration, applying bronchial blocking devices. The current research investigated the advantages of thoracoscopic-assisted oesophagectomy for oesophageal cancer, which was conducted under right bronchial occlusion with one-lung ventilation in the prone position.

## 2 Methods

### 2.1 Patients and data collection

This study gathered 105 thoracoscopic-assisted oesophagectomy anaesthesia records from the same surgeon *via* the Anaesthesia Information Management System (AIMS) from July 2020 to June 2021. The patients underwent oesophageal surgery in the prone position (n = 43) or the semiprone position (n = 62).

#### 2.1.1 Inclusion and exclusion criteria

Inclusion criteria were ASA II–III grade scheduling for artificial pneumothorax under right bronchial occlusion, one-lung ventilation, thoracoscopic-assisted oesophagectomy in the prone position or the semiprone position. The choice of the prone position or the semiprone position depends on the preference of the surgeon. Conversion to open thoracotomy or laparotomy, total laryngectomy, lung resection, and reoperation were excluded.

Ultimately, 103 patients with thoracoscopic-assisted oesophagectomy were included in the final analysis, including 43 patients in the prone position.

### 2.2 Preoperative preparations and anaesthesia protocol

No premedication was administered to the patients. In the operating room, the patients were monitored with noninvasive blood pressure (NIBP), bispectral index (BIS), pulse oximetry, electrocardiography (ECG), and right internal jugular central venous catheterization (CVC). The patients were injected with crystalloid (6 ml/kg) *via* a catheter inserted into a peripheral vein. Invasive blood pressure was monitored through cannulation of the radial artery after local administration of lidocaine anaesthesia. A target-controlled infusion (TCI) of 2% propofol was adopted to induce anaesthesia at an effect-site concentration (Ce) of 4 μg/ml, sufentanil at 0.6 μg/kg, cisatracurium at 0.2 mg/kg, and dexmedetomidine (DEX) at 1 μg/kg for 10 min; patients were intubated with a single-lumen endotracheal tube applying bronchial blockers by senior thoracic anaesthesiologists taking part in the research, and a fibreoptic bronchoscope (FOB) to confirm the correct location. During the periods of two-lung ventilation (TLV) and one-lung ventilation (OLV), a tidal volume of 7 ml/kg, a respiratory rate of 12 bpm, and an I/E proportion of 1:2 were realized. Cisatracurium at 0.12 mg/kg/h and 2% propofol Ce at 2–3 μg/ml titrated were used to maintain anaesthesia to keep BIS between 40 and 50; the average arterial blood pressure (MAP) and heart rate (HR) were 20% lower than the baseline values. The placement of the patient in a lateral semiprone position or prone position done after right internal jugular central venous catheterization. A FOB was adopted to confirm the correct bronchial blocker location. Then, 100% oxygen was used to initiate and maintain anaesthesia induction and one-lung ventilation (OLV). The pressure of the artificial pneumothorax was 8 mmHg, and the flow of the artificial pneumothorax was 8 L/min. At the end of the procedure, the chest was closed, the bronchial blocker was removed, the lung was restored with a manoeuvre, and the inspiratory pressure was increased to 40 cmH_2_O. Fifty percent oxygen and 5 cm H_2_O of positive end expiratory pressure (PEEP) were adopted to maintain two-lung ventilation. While completing the operation, an electronic infusion pump (FSQ-11 PCA; Inc., JiangSu AIPENG, ED, China) for patient-controlled anaesthesia (PCA) was set up for each patient. After surgery, patients are transported to the post-anaesthetic care unit (PACU).

### 2.3 Measurements

Baseline demographic and clinical data were collected, including age, sex, BMI, ASA grade, hypertension, diabetes, coronary heart disease, stroke, radiotherapy/chemotherapy, FEV1/FVC, DLCO, and respiratory comorbidity. The intraoperative and post-operative variables, such as total operative time, chest tube duration, total fluid volume, total blood loss, intraoperative blood transfusion, urine volume, intraoperative hypoxemia (SpO_2_ <90%), conversion to open thoracotomy, length of stay in the PACU, and PCA pressing frequency in 24 h, were collected too.

#### 2.3.1 Primary outcome

The primary endpoint measures were PPCs (atelectasis, bronchospasm, aspiration pneumonitis, pulmonary infection, and respiratory failure). Radiologists measured atelectasis by chest X-ray (CXR) within 3 days after the operation. Respiratory failure and pulmonary infection were assessed with reference to the European Perioperative Clinical Outcome (EPCO) (14). All PPCs were evaluated within 7 days of the operation.

#### 2.3.2 Secondary outcomes

The secondary endpoint measures were reintubation, anastomotic leakage, chylothorax, surgical site infection, atrial arrhythmia, acute cerebral infarction, myocardial infarction, reoperation, recurrent laryngeal nerve palsy, chest tube duration, postoperative length of stay, and ICU stay.

### 2.4 Statistical analysis

Quantitative variables are shown as the mean ± standard deviation. Categorical variables are presented as frequencies and fractions. SPSS version 25.0 (SPSS, Chicago, IL) was used to perform the statistical research. The comparison of all categorical variables was made with the χ^2^ test or Fisher’s exact test. The nonparametric Mann–Whitney U test or t-test was adopted to test continuous variables with a nonnormal distribution. Atelectasis was significantly associated with all risk factors by univariate analysis and was entered into a multivariable logistic regression model using a forward (LR) selection strategy. ROC analysis was used to determine the thresholds for DLCO% and surgery time. Two-sided p values <0.05 were considered indicative of statistical significance.

## 3 Results

**Figure 1:**
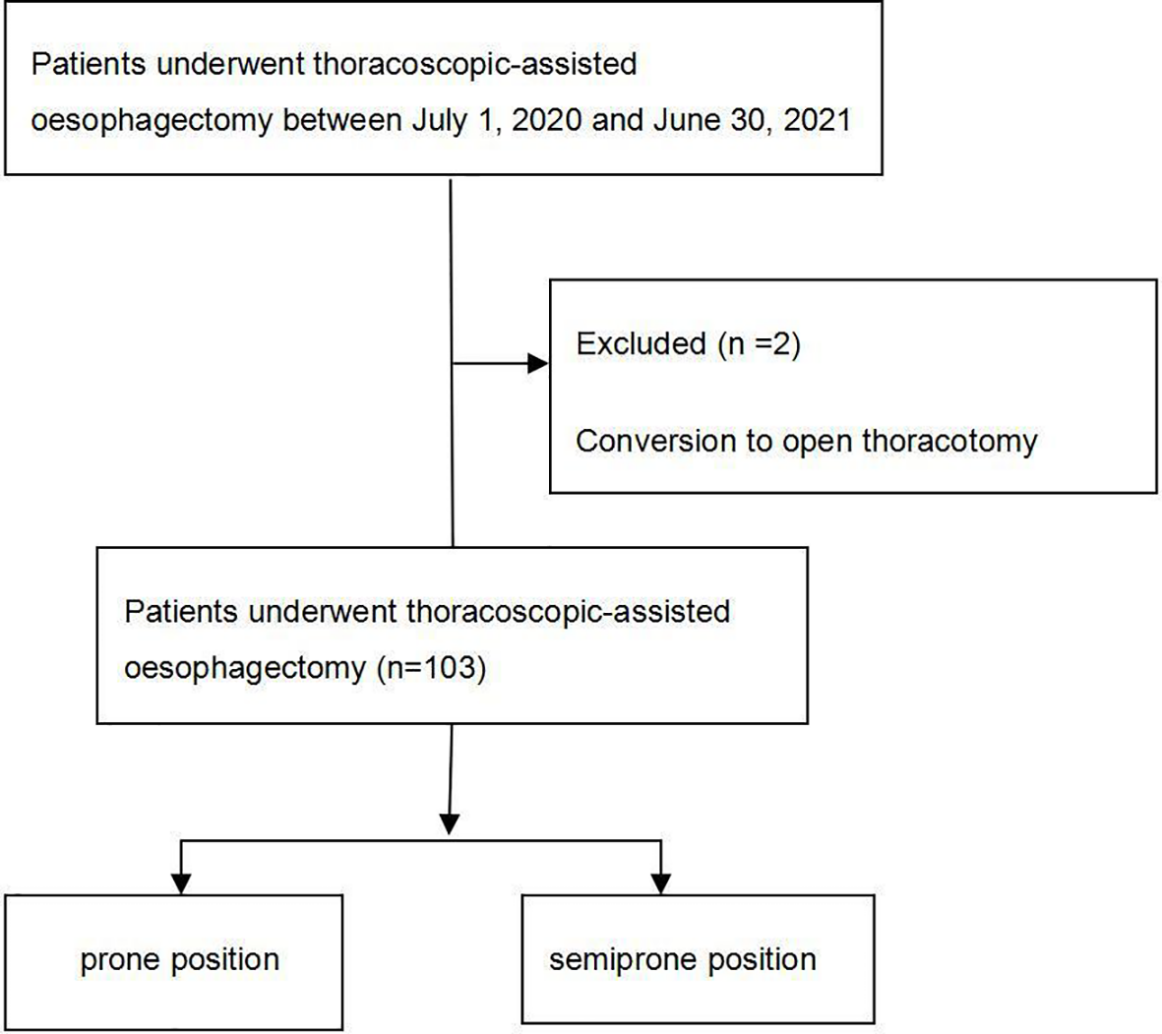
Consort flow diagram.

There were 105 patients in this research, but two were excluded. The final analysis (**Figure 1**) used information about 103 patients. No significant differences were found between the demographic data of the groups (**Table 1**), namely age, sex, BMI, ASA grade, hypertension, diabetes, coronary heart disease, stroke, radiotherapy/chemotherapy, FEV1/FVC, DLCO, and respiratory comorbidity (P >0.05, **Table 1**).

**Table 1:** Preoperative patient characteristics.

Variable	Prone position (n = 43)	Semiprone position (n = 60)	P-value
Age (years)	65.4 ± 6.2	67.5 ± 6.4	0.105
Sex			0.448
Male	33 (76.7)	42 (70.0)	
Female	10 (23.3)	18 (30.0)	
BMI (kg/m^2^)	22.9 ± 3.0	22.8 ± 3.0	0.893
ASA grade			0.503
II	32 (74.4)	41 (68.3)	
III	11 (25.6)	19 (31.7)	
Hypertension	16 (37.2)	25 (41.7)	0.649
Diabetes	15 (34.9)	24 (40.0)	0.598
Coronary heart disease	8 (18.6)	15 (25.0)	0.442
Stroke	5 (11.6)	11 (18.3)	0.354
COPD	1 (2.3)	2 (3.3)	0.624
Interstitial lung disease	0 (0)	0 (0)	/
Pneumonia	2 (4.7)	5 (8.3)	0.696
Radiotherapy/chemotherapy	10 (23.3)	18 (30.0)	0.448
FEV_1_/FVC, %	102.2 ± 7.2	100.2 ± 11.4	0.313
DLCO%	94.5 ± 15.3	93.5 ± 18.6	0.773
Smoking habit (yes/no)	16 (37.2)	14 (23.3)	0.096
Respiratory comorbidity (yes/no)	3 (7.0)	7 (11.7)	0.515

BMI, Body mass index; ASA, American Society of Anesthesiologists; COPD, Chronic obstructive pulmonary disease; FEV_1_, Forced expiratory volume in the first second; FVC, Forced vital capacity; DLCO, Carbon monoxide diffusing capacity.

As shown in **Table 2**, patients in the prone position had a longer total operative time (260.1 ± 44.4 min vs 241.2 ± 49.1 min, P = 0.048). The other intraoperative surgical outcomes did not differ significantly. The patients in the prone position had a decreased incidence of atelectasis (12% vs. 30%, P = 0.032) (p <0.05, **Table 3**). No significant differences in the rates of cardiovascular and other complications, length of ICU stay, or LOS were found between the two groups (P >0.05) (**Table 4**).

**Table 2:** Intraoperative surgical outcomes.

Variable	Prone position (n = 43)	Semiprone position (n = 60)	P-value
Total operative time, min	260.1 ± 44.4	241.2 ± 49.1	0.048
Chest part, min	110.2 ± 24.2	98.1 ± 30.3	0.033
Total fluid volume, ml	2,132.6 ± 463.8	1,970.0 ± 370.6	0.051
Total blood loss, ml	196.5 ± 75.9	194.2 ± 79.2	0.881
Intraoperative blood transfusion (Yes/No)			0.624
Yes	1 (2.3)	2 (3.3)	
No	42 (97.7)	58 (96.7)	
Urine volume, ml	405.3 ± 236.5	329.5 ± 206.2	0.087
Intraoperative hypoxemia	11 (25.6)	20 (33.3)	0.514
Duration in PACU, min	34.7 ± 13.3	39.5 ± 13.0	0.069
PCA pressing frequency in 24 h	7.8 ± 4.8	8.0 ± 4.7	0.839

PACU, Postanaesthetic care unit; PCA, Patient-controlled analgesia.

**Table 3:** Postoperative pulmonary complications between the prone position and semiprone position group.

Variable	Prone position (n = 43)	Semiprone position (n = 60)	P-value
Pulmonary infection	5 (11.6)	11 (18.3)	0.354
Respiratory failure	1 (2.3)	1 (1.7)	0.663
Atelectasis	5 (11.6)	18 (30.0)	0.032
Bronchospasm	2 (4.7)	5 (8.3)	0.696
Aspiration pneumonitis	8 (18.6)	12 (20.0)	0.534

**Table 4:** The other postoperative complications between the prone position and semiprone position.

Variable	Prone position (n = 43)	Semiprone position (n = 60)	P-value
Reintubation	1 (2.3)	1 (1.7)	0.663
Anastomotic leakage	1 (2.3)	2 (3.3)	0.624
Chylothorax	1 (2.3)	1 (1.7)	0.663
Surgical site infection	1 (2.3)	0 (0.0)	0.417
Atrial arrhythmia	3 (7.0)	4 (6.7)	0.623
Acute cerebral infarction	1 (2.3)	1 (1.7)	0.663
Myocardial infarction	0 (0.0)	1 (1.7)	0.583
Reoperation	0 (0.0)	1 (1.7)	0.583
Recurrent laryngeal nerve palsy	8 (18.6)	10 (16.7)	0.798
Chest tube duration, days	5.0 ± 6.5	4.3 ± 2.0	0.426
Postoperative length of stay, days	15.4 ± 7.6	15.5 ± 4.3	0.891
ICU stay, days	2.7 ± 3.3	2.3 ± 2.6	0.479

ICU, Intensive care unit; BMI, Body mass index.

Multivariable logistic regression analysis showed that the prone position (OR = 0.196, P = 0.011), smoking (OR = 0.103, P <0.001), preoperative DLCO% ≥90% (OR = 0.230, P = 0.003), and an operative time <180 min (OR = 0.268, P = 0.006) were associated with less atelectasis (**Table 5**).

**Table 5:** The results of the bivariate and multivariate analyses of the factors associated with atelectasis.

Variable	Atelectasis		Univariate	Multivariate	
	Yes (n = 23)	No (n = 80)	P-value	OR (95% CI)	P-value
Position			0.027		
Prone position	5	38		0.196 (0.055–0.692)	0.011
Semiprone position	18	42			
Smoking habit	19	4	<0.001	0.103 (0.032–0.334)	<0.001
DLCO%			0.045		
<90%(reference)	16	65		0.230 (0.054–0.971)	0.003
≥90%	7	15			
BMI (kg/m^2^)	23.6 ± 2.1	22.0 ± 3.2	0.019		
Intrahypoxemia	22	11	<0.001		
Operative time (min)			0.385		
≥180	17	30		0.268 (0.103–0.528)	0.006
<180	6	50			

DLCO, Carbon monoxide diffusing capacity.

## 4 Discussion

Our study showed that artificial pneumothorax under one-lung ventilation for patients with thoracoscopic-assisted oesophagectomy in the prone position was related to fewer PPCs than that in the semiprone position. The prone position could reduce the rate of atelectasis. Nevertheless, according to the results, in thoracoscopic-assisted oesophagectomy, the prone position has no effect on cardiovascular and other complications, ICU stay length and LOS. Therefore, in terms of PPCs, thoracoscopic-assisted oesophagectomy in the prone position may help to provide patients with more benefits.

It is not surprising that approximately 22.3% (23/103) of patients scheduled for thoracoscopic-assisted oesophagectomy have possible PPCs given the same prevalence of PPCs in other clinical studies (7). For example, recent studies have reported postoperative pulmonary complication rates of 19%–32% and in-hospital mortality rates ranging from 2 to 6% (7). The definition of PPCs in this research is based on the European Perioperative Clinical Outcome definition (14). From a surgical perspective, the operative field perspective in the prone position without compression of the right lung is improved by artificial pneumothorax and gravity, leading to no mechanical damage to the lungs. This technique has considerable merits, including enhanced surgeon ergonomics, increased operative field exposure, and excellent respiratory outcomes (15). Thoracoscopic-assisted oesophagectomy in the prone position is related to better surgical ergonomics compared with that in the semiprone position because of gravity pooling blood outside the operative field and the decreased demand for lung retraction (16).

A higher risk of airway secretions flowing into the left lung will be observed if the patient is placed in the semiprone position compared with the prone position. The enhancement in oxygenation may be one of the causes of reduced atelectasis in the prone position. The prone position could stop recurrent nerve palsy due to the wider view (17).

Thoracoscopic-assisted oesophagectomy in the prone position was related to fewer PPCs and decreased mortality in patients compared with that in the semiprone position (7). Therefore, this study confirms that PPCs could be reduced by thoracoscopic-assisted oesophagectomy in the prone position as opposed to the semiprone position. On the basis of this research, no great differences were shown in the rates of other postoperative complications, ICU stay length, or LOS.

It was shown that the prone position improves oxygenation in different diseases, including acute respiratory distress syndrome, pneumonia, and atelectasis (18–21). Physiologically, patients lying in the prone position have a satisfactory functional residual capacity (FRC) and ventilation/perfusion proportion. The prone position did not greatly influence the respiratory system or lung and chest wall compliance but improved lung volumes and oxygenation during general anaesthesia (22). FRC is reduced in the semiprone position because of increased intra-abdominal pressure and exclusion from the mediastinum compared with the supine position. In contrast, the FRC is increased in the prone position because of reduced cephalad pressure on the diaphragm (3). In the current research, there was mild variation in the ventilation/perfusion proportion in the prone position; alternatively, the decline in the ventilation/perfusion proportion in the semiprone position may be caused by increased blood flow to the left lung because of gravity. Moreover, while increasing the constriction of lung blood vessels, a higher carbon dioxide partial pressure (pCO_2_) caused by artificial CO_2_ pneumothorax enhances the ventilation/perfusion proportion in the prone position (23, 24).

There are several limitations to this research. First, the analyses on the basis of administrative coding data may be susceptible to reporting bias or coding mistakes. Second, the patients were not randomized into receiving artificial pneumothorax under right bronchial occlusion one-lung ventilation with thoracoscopic-assisted oesophagectomy in the prone position or the semiprone position. Future studies, such as randomized trials, may be indispensable for a more precise discussion to compensate for selection bias.

In conclusion, our study shows that artificial pneumothorax under right bronchial occlusion one-lung ventilation for patients with thoracoscopic-assisted oesophagectomy in the prone position may decrease PPCs by decreasing atelectasis in patients undergoing thoracic surgery compared with those in the semiprone position.

## Data availability statement

The raw data supporting the conclusions of this article will be made available by the authors, without undue reservation.

## Author contributions

QL: This author helped design and writing original draft preparation. MZ: This author helped data analysis and collection and manuscript writing. DW: This author conceptualization, methodology, and manuscript writing. XG: This author helped data collection, analysis and manuscript critical revision. JW: This author helped conceptualization, methodology, administrative support, manuscript writing, reviewing and editing. All authors listed have made a substantial, direct, and intellectual contribution to the work and approved it for publication.

## Funding

This work was supported by the Shanghai Shen Kang Hospital Development Center Project (SHDC2020CR4063) and the Nurturing Projects for Basic Research of Shanghai Chest Hospital (2021YNJCQ10) & 2019YNJCM08.

## Conflict of interest

The authors declare that the research was conducted in the absence of any commercial or financial relationships that could be construed as a potential conflict of interest.

## Publisher’s note

All claims expressed in this article are solely those of the authors and do not necessarily represent those of their affiliated organizations, or those of the publisher, the editors and the reviewers. Any product that may be evaluated in this article, or claim that may be made by its manufacturer, is not guaranteed or endorsed by the publisher.
